# Vertex models: from cell mechanics to tissue morphogenesis

**DOI:** 10.1098/rstb.2015.0520

**Published:** 2017-03-27

**Authors:** Silvanus Alt, Poulami Ganguly, Guillaume Salbreux

**Affiliations:** The Francis Crick Institute, 1 Midland Road, London NW1 1AT, UK

**Keywords:** vertex models, tissue mechanics, epithelial mechanics, morphogenesis, simulations

## Abstract

Tissue morphogenesis requires the collective, coordinated motion and deformation of a large number of cells. Vertex model simulations for tissue mechanics have been developed to bridge the scales between force generation at the cellular level and tissue deformation and flows. We review here various formulations of vertex models that have been proposed for describing tissues in two and three dimensions. We discuss a generic formulation using a virtual work differential, and we review applications of vertex models to biological morphogenetic processes. We also highlight recent efforts to obtain continuum theories of tissue mechanics, which are effective, coarse-grained descriptions of vertex models.

This article is part of the themed issue ‘Systems morphodynamics: understanding the development of tissue hardware’.

## 1. Introduction

**Figure 1.**
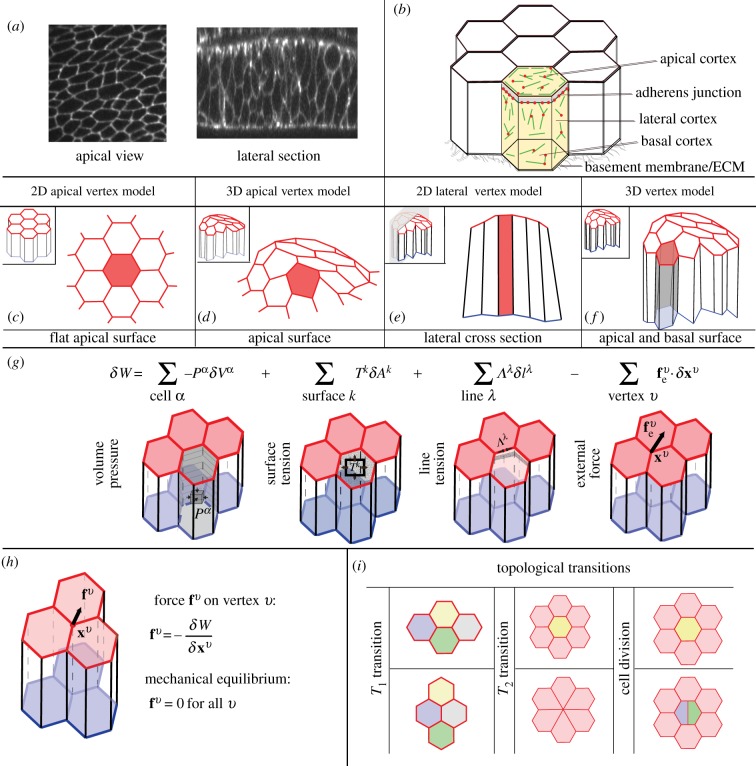
Properties of 2D and 3D vertex models for tissue mechanics. (*a*) Apical view (from [1]) and cross-section (from [2]) of the wing imaginal disc epithelium in the *Drosophila* embryo. (*b*) Schematic of an epithelial tissue. Cytoskeletal elements generate forces inside the cells, which are mechanically coupled to other cells and to the basement membrane. (*c*,*d*) In apical vertex models epithelial cells are represented by the shape of their apical surfaces, which are polygons either in 2D or in 3D. (*e*) In 2D lateral vertex models, cells are represented by their lateral cross-sections. (*f*) In 3D vertex models the tissue is represented by its apical and basal geometry. (*g*) A virtual work differential for vertex displacement, depending on changes in cell volume *δV*^*α*^, surface area *δA*^*k*^ and edge length *δl*^λ^. External forces acting on vertices 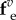 can yield an additional contribution to the virtual work. (*h*) The force **f**^*v*^ on a vertex *v* is obtained by taking the virtual work differential with respect to the vertex position **x**^*v*^. The tissue is in mechanical equilibrium when the force acting on all vertices is zero. (*i*) Topological transitions in epithelia are cell–cell intercalations (*T*_1_ transitions), cell extrusions (*T*_2_ transitions) and cell divisions.

Cells in tissues are mechanically coupled to their neighbours by adhesion molecules along their common interfaces (figure 1*a*,*b*), and exert forces onto each other and on their environment. These complex interactions can lead to significant morphogenetic deformations of developing tissues, such as folding, stretching or constriction, which are crucial to setting the shape of the organism. Understanding how cells collectively achieve this task is a major question at the interface of physics and developmental biology.

Internal cellular stresses lead to effective forces that act to deform the cell shape. The cytoskeleton plays a major role in generating such internal stresses [3,4]. The actin cortex in particular generates surface tensions and line tensions acting along the cell membranes, which are mediated and counterbalanced by cell–cell and cell–extracellular matrix adhesion [5–8] (figure 1*b*). Both during development and in the adult stage, tissues are additionally subjected to mechanical forces exerted by the surrounding environment. External structures such as the basement membrane [2,9] or the apical extracellular membrane [10,11] can mechanically constrain tissues.

Tissue morphogenesis occurs when mechanical properties of the cells are changed, when cells divide or undergo apoptosis, or when physical constraints imposed by the environment onto the epithelium are altered. Understanding the physics of morphogenesis requires taking into account the laws of mechanics, which imply that forces acting in a tissue have to be balanced. Internal and external forces that act to deform the tissue are balanced by friction or viscous forces.

Quantitative physical descriptions of tissues enable a description of how generation and balance of mechanical forces drive morphogenesis [12–14]. Several approaches have been proposed to describe the physics of epithelia [15,16], a class of tissue where cells are arranged in a nearly two-dimensional (2D) structure (figure 1*a*). Here, we discuss vertex models, which describe epithelia by a set of vertices [17,18]. Vertex models have been developed to understand how the complex interplay between cellular shape, the forces generated inside epithelial cells and mechanical constraints externally imposed on the tissue act together to drive tissue morphogenesis. In this review, we discuss various vertex models that have been proposed, and describe their mechanical formulation in a general form. We summarize how previous applications of vertex models have helped us to understand biological processes, and conclude by discussing the link between vertex models and coarse-grained, continuum description of tissues.

## 2. Vertex models for tissue mechanics

### (a) Geometry of vertex models

In vertex models, the epithelial shape is represented by a set of vertices that mark the common point of three or more neighbouring cells. Cellular interfaces and cell volumes can be defined from the positions of the vertices. We suggest classifying vertex models into four groups, depending on their geometrical representation of tissues: 2D and three-dimensional (3D) apical vertex models, 2D lateral vertex models and 3D vertex models.

#### (i) 2D apical vertex models

Vertex models were initially developed to study the packing of bubbles in foams [19,20]. This approach was then adapted to study the 2D packing and rearrangement of apical cell surfaces in planar epithelia [1,21–30]. In these studies, epithelia are described by a planar 2D network of vertices that defines the apical cell surfaces as polygons with straight interfaces between neighbouring cells (figure 1*c*). The underlying assumption of 2D apical vertex models is that the main forces acting to deform the cells are generated along the apical cell surfaces, or can be effectively absorbed in the apical representation.

#### (ii) 3D apical vertex models

In order to describe folding epithelia, 2D apical vertex models were extended by representing the apical tissue surface as a 2D manifold in 3D space [31–34] (figure 1*d*). To allow for non-planar tissue configurations, apical cell surfaces are not considered to be flat polygons. Instead, vertices can lie on a fixed manifold, or apical membranes can be represented by triangulated surfaces, where each bond forms a triangle with the midpoint of the apical cell surface. This approach relies on the idea that the tissue mechanics can be effectively described by the apical cell surfaces only, and therefore that the configuration and shape of the basal part of the tissue does not play a significant mechanical role.

#### (iii) 2D lateral vertex models

2D lateral vertex models describe a planar cross-section of an epithelium (figure 1*e*). Cells are quadrilaterals, with two edges representing the apical and basal section of the tissue, and two edges representing lateral interfaces connecting a cell to its neighbours [35–40]. The underlying assumption of 2D lateral vertex models is that the tissue shape does not vary significantly in the direction perpendicular to the plane of the cross-section.

#### (iv) 3D vertex models

In 3D vertex models, cells are polyhedrons that are described by vertices in 3D space [2,41–44] (figure 1*f*), and all cells are enclosed by triangulated surfaces. In 3D vertex models for epithelia, each cell has distinct apical and basal surfaces, and is connected to its neighbours by lateral interfaces [2,43–45]. In [2,44], the apical and basal networks of vertices have the same topology, such that two cells are connected basally if they are connected apically. This constraint has been released in [41,43,45], for instance, to allow cell delamination to be described or allow for neighbour exchange along the apico-basal axis, which may occur in highly columnar tissues. 3D vertex models have also been applied to describe three-dimensional cell aggregates where cells have neighbours in all directions of space [41,43,45,46].

### (b) Mechanical description

To determine the motion of vertices, mechanical forces must be specified. We discuss below a formulation of forces generated in vertex models capturing general aspects of previously proposed vertex model descriptions. Instead of introducing an effective energy depending on the vertex positions, we suggest a more general virtual work formulation, which holds for arbitrary dependencies of pressures and tensions on the geometry.

#### (i) Virtual work function

Cells of a tissue are labelled with an index *α*, apical and basal surfaces and lateral interfaces with the index *k*, edges are labelled by λ and vertices are labelled *v*. Each vertex *v* has a position **x**^*v*^ which reads 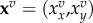 or 
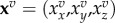
 in cartesian coordinates in 2D and 3D, respectively. The internally generated forces in the model can be obtained from an internal virtual work differential *δW*_*i*_. Changes in internal virtual work *δW*_*i*_ can, for instance, result from changes in the cell volumes *δV*^*α*^, in the areas of surfaces *δA*^*k*^ or in the lengths of bonds *δl*^λ^. By defining the cell pressure *P*^*α*^, the surface tensions *T*^*k*^ and the line tensions *Λ*^λ^, the differential of the internal virtual work for vertex movements can be written as
2.1
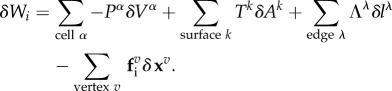
The geometrical quantities *V*^*α*^, *A*^*k*^ and *l*^λ^ and the mechanical properties *P*^*α*^, *T*^*k*^ and *Λ*^λ^ are functions of the current shape of the tissue characterized by the vertex positions **x**^*v*^. The mechanical properties can, in general, also depend on time. The last term in equation (2.1) is a generic force acting on the vertices that allows capture of additional terms, such as the bending elasticity of surfaces [33] or a tension acting to change the cell height [34]. To illustrate the relation between the virtual differential work (2.1) and effective energy functions, we consider the function of [22]
2.2
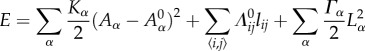
with *L*_*α*_ the perimeter of cell *α*. By differentiation of the energy function with respect to the cell area *A*_*α*_, one then obtains the surface tension of cell *α*, 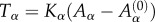. The line tension of the edge joining vertices *i* and *j* is given by 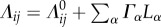, where the sum runs over cells that are neighbours to the edge. The line tension has two contributions: one is independent of the edge length, while the second one is an elastic term related to the cell perimeter.

In addition to the internal virtual work, which characterizes forces generated inside the cells, external forces acting onto the epithelium can be included in an external virtual work *δW*_e_. External forces can arise from external compression or tension applied to the tissue [2,47,48], fluid pressure acting on apical or basal cell surfaces [37,44], or attachment of cells to the underlying basement membrane [2]. The external virtual work can be written as
2.3
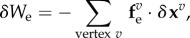
where the external forces 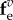 vary in different models. The total differential of the virtual work is the sum of the internal and external contributions (figure 1*g*),
2.4
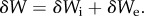


#### (ii) Forces acting on vertices

Forces acting on vertices can be derived from the virtual differential work (2.4) by differentiating the virtual work with respect to vertex positions (figure 1*h*)
2.5
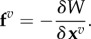
A tissue configuration is a stable mechanical equilibrium if the forces on all vertices vanish, **f**^*v*^ = 0, and any small deviation from the configuration results in a restoring force, i.e. the force gradient ∂**f**^*v*^/∂**x**^*v*′^ is a negative definite matrix.

#### (iii) Tissue dynamics

Morphogenetic processes involve the deformation of tissues in time, with cells showing highly dynamic behaviour [49]. Dynamics of vertex motion must be included in order to reproduce the time evolution of the tissue shape changes. Several approaches have been proposed to describe the dynamics of vertices. In the quasi-static approach, the tissue is assumed to relax instantly to the closest mechanical equilibrium state after each perturbation of the tissue [1,2,22,25,30]. The timescale of tissue deformation is therefore set by temporal variations of the tissue mechanical parameters. The relaxation to the closest mechanical equilibrium state can be implemented using high-dimensional minimization methods, such as the gradient or the conjugate gradient method. The definition of ‘closest’ depends on the method used to identify the subsequent minima, and if several minima of the work function exist different minimization methods might lead to different local minima and thus induce different quasi-static dynamics.

Alternative approaches include explicitly dissipative processes that limit the motion of vertices. Dissipative processes can be introduced by writing a force balance equation for vertex *v* including viscous terms
2.6
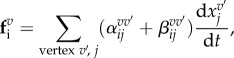
where *i*, *j* denote spatial coordinates. Although static and dissipative forces are distinguished in equation (2.6) for this discussion, both can be included in the pressure, tensions and forces of equations (2.1) and (2.3). A distinction can be made between external sources of dissipation arising from interactions with the environment in which cells are moving, and internal dissipation within the tissue: 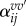 is a friction matrix associated with external dissipative processes, such as friction against an external substrate, and 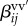 is associated with internal dissipative processes, related to the resistance to deformation of epithelial structures such as bonds, surfaces or cell volumes [35,42,43,50,51]. Owing to Galilean invariance, when dissipation is purely internal, the friction matrix ***β*** must satisfy 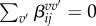. The friction matrices depend in general on the current geometry of the tissue. Solving the first order differential system given by equation (2.6) requires the friction matrix to be invertible [51]. If the matrix is not invertible, indicating that some modes of deformations relax infinitely fast, additional dissipative processes must be taken into account. Equation (2.6) is sometimes simplified to an effective drag force acting on vertex *v* with coefficient *α*_*v*_ [21,35]
2.7
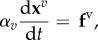
which is akin to an external source of dissipation. Little is known from the experimental point of view on the dominating dissipative processes in an epithelium on the timescales of minutes to hours generally considered by vertex models. Possibly, rheological experiments where a controlled force is applied to a tissue and the tissue deformation is monitored over time could be used to estimate the magnitude of the friction matrices introduced above [48]. Effective friction provided by transient binding to the extracellular-matrix, viscous flows induced in the cytoskeleton, or junction remodelling may be possible sources of dissipation entering the determination of these coefficients.

### (c) Topological transitions

In the course of epithelial morphogenesis, cells do not only change their shapes, but also divide, can be extruded from the tissue and change their neighbours [6]. These topological transitions have been introduced in the framework of vertex model simulations [19,21,22,42]. Neighbour exchange is usually implemented by defining a minimal length below which two threefold vertices merge into a fourfold vertex, which then opens again to form an edge between two previously unconnected cells (figure 1*i*). The minimal length has been chosen to be around 5–20% of a characteristic length set by the square root of the cell reference area [21,24,42].

Apoptosis and delamination of cells in developing and adult tissues can be triggered cell autonomously, or by chemical or mechanical cues [52–55]. To represent cell extrusions in vertex models, the edges of a cell are replaced by a single vertex at the centroid of the cell (figure 1*i*). Cells are extruded if their surface area shrinks below a predefined threshold [52,56,57], or if another apoptotic signal triggers their removal [34]. Cell divisions in epithelia are simulated in vertex models by introducing new vertices, and dividing the cell with a new edge in apical vertex models [22], or with a new lateral interface in 3D vertex models [2,43,58] (figure 1*i*). Different models of cell growth and division have been used. In [22], the preferred cell area of a dividing cell is doubled quasi-statically, a new randomly oriented edge is introduced, and the two daughter cells are assigned the original properties of the mother cell. A division algorithm which does not involve quasi-static doubling of the preferred area prior to division yields qualitatively the same results [1]. Different rules for orienting cell divisions have been explored and compared with experimental data. Cell divisions have, for instance, been chosen to orient preferentially in the direction of cell elongation or growth [30,43], or a random choice of division plane orientation in isotropically growing cells has also been considered [59,60].

Finally, additional topological transitions are, in principle, possible; a topological transition where a vertex meets and consequently fuses with an edge has been introduced, for instance, in the context of wound healing [24].

## 3. Applications of vertex models

We now discuss previous applications of vertex models to morphogenetic processes.

### (a) 2D apical vertex models

Apical vertex models have been used extensively to characterize the cell packing topology in epithelia. Quantitative comparisons of the distribution of cell areas and of number of cell neighbours in vertex model simulations and experiments have been performed in growing epithelial tissues in *Drosophila*, *Xenopus*, *Hydra* and the plant *Arabidopsis* [1,22,60–62]. In most of these studies, the cell packing results from a sequence of cell divisions of randomly chosen cells, in between which the cell packing is relaxed quasi-statically. The introduction of a perimeter elasticity term to the virtual work function in equation (2.1) allows for parameter configurations where the vertex model ground states correspond to an irregular soft network, instead of a regular honeycomb packing [1,22]. The experimentally observed cell shapes in the *Drosophila* wing disc suggest, however, that the tissue is within the parameter regime where the hexagonal regular packing is the ground state. A transition between liquid and solid regimes has been characterized in a simulated tissue where vertex model parameters are varied [63], and has been suggested to occur in biological systems such as the asthmatic airway epithelium [64].

Apical vertex models have been a tool of choice to study tissue growth. The effect of mechanical feedback on tissue growth [23,28,61], and the influence of differential rates of growth on the cell packing [65] have been investigated using these models. To study the effects of signalling on tissue growth, a number of morphogen molecules per cell has been introduced in the vertex models that evolves over time according to production, degradation and transport [27,28,66]. Tissue homeostasis and the balance between cell division and cell extrusion have also been studied in vertex model frameworks [52].

Planar cell polarity pathways, which allow cells to establish an anisotropic distribution of polarity proteins in the plane of the tissue, have been considered in vertex models by assigning a value to edges representing the density of planar polarity proteins [26,29]. Reorientation of planar cell polarity by tissue flows can be understood with such a description [26]. A model where the line tension of interfaces is regulated by a cell planar polarity pathway has been proposed to describe the regular arrangement of cone photoreceptors on a rectangular lattice in the zebrafish retina [29].

**Figure 2.**
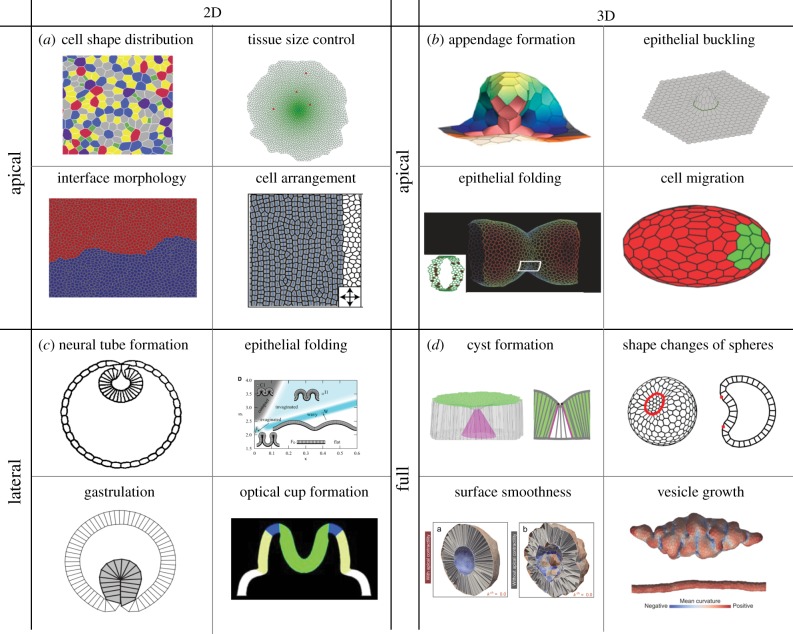
Examples of biological processes described by vertex models. (*a*) 2D apical vertex models have been used to study cellular arrangements in planar epithelia. (*top left*) Cell packing topology in growing epithelia [1,22,60–62]. The image shows a simulated epithelium where cells are coloured depending on their neighbour number (from [22]). (*top right*) Epithelial growth and size control [23,27,28,61,66]. The image shows the final shape of a growing epithelium with a morphogen concentration indicated in shades of green (from [23]). (*bottom left*) Interface smoothing between differently fated epithelia due to differential tension [25,30,67]. The image shows a simulation of the boundary between two compartments (red and blue) of the wing imaginal disc in *Drosophila* (from [25]). (*bottom right*) Simulation of cone photoreceptor packing in the zebrafish retina, showing ordering of cells due to polarized interfacial stresses (from [29]). (*b*) 3D apical vertex models for epithelia where the apical surfaces of cells move out of plane, and stresses are generated along the apical cell surfaces. (*top left*) Simulation of appendage formation on the *Drosophila* eggshell due to mechanical patterning (from [32]). (*top right*) Theoretical study of the buckling of a compressed epithelium (from [33]). (*bottom left*) Simulation showing epithelial folding due to apoptotic forces in the imaginal leg disc of *Drosophila* (from [34]). (*bottom right*) Simulation of anterior visceral endoderm migration (green) among the visceral endoderm (red) in the egg-cylinder stage mouse embryo (from [31]). (*c*) 2D lateral vertex models describe a cross-section of the epithelium. (*top left*) Simulation of neural tube formation in amphibians as a result of a ‘purse-string’ contraction of apical surfaces and cell volume conservation (from [35]). (*top right*) Phase diagram of epithelial buckling as a function of bending stiffness and differential tensions along the apical and basal surfaces of cells (from [40]). (*bottom left*) Simulation of ventral furrow formation in *Drosophila* (from [37]). (*bottom right*) Simulation of optical cup formation in mouse embryonic stem cell culture (from [36]). (*d*) 3D vertex models have been used to study deformations of epithelia in three dimensions taking into account stresses along apical, basal and lateral surfaces. (*top left*) Cyst formation in the *Drosophila* imaginal wing disc due to a contractile boundary; 3D geometry of the cells with an invaginating cyst in the centre (left) and a cross-section through the cyst (right) (from [2]). (*top right*) Deformations of a patterned spherical epithelia (from [44]). (*bottom left*) Simulation results of proliferating tissue with (left) and without (right) apical contractility, showing that apical and basal smoothness are increased with apical contractility (from [45]). (*bottom right*) Simulation of growth of epithelial vesicles, with different choices for viscous dissipation (from [43]).

Apical vertex models have also been used to study the effect of differential tension between tissues. The roughness of the boundaries between compartments of the fly wing disc has been analysed with a vertex model involving differences in interfacial tensions [25,30] (figure 2*a*), possibly driven by signalling molecules [67]. An increase in line tensions at the interface between two growing neighbouring tissues tends to maintain a smooth boundary, while internal fluctuations arising through random cell divisions tend to favour a rough boundary. The process of wound healing in *Xenopus* embryos, where cells collectively migrate to close a hole, has also been analysed in terms of surface and interfacial tensions in a vertex model [24]. On the scale of a few cells, the regular arrangement of cells in the retina of the *Drosophila* eye has been studied in a vertex model that allows for the curvature of edges [68] (figure 2*a*).

### (b) 3D apical vertex models

3D apical vertex models, where vertices can move in three dimensions, have been introduced to capture morphogenetic processes where an epithelium deforms out of plane. During the morphogenesis of the respiratory appendages on eggshells of *Drosophila*, a 2D pattern of line tensions along cell edges results in tissue buckling that resembles experimentally observed deformations [32] (figure 2*b*). The buckling of a flat epithelium in response to compression has been explored using a 3D apical vertex model with bending elasticity [33] (figure 2*b*). Simulations of the fold formation in the roughly cylindrical leg imaginal disc in *Drosophila* have been performed to support the idea that contractile forces generated by cells undergoing controlled apoptosis drive fold formation [34] (figure 2*b*). Cell migration has also been introduced by adding an external force acting on some vertices, driving the cell motion, and the resulting packing perturbation has been studied in relation to cell migration in mouse embryos [31] (figure 2*b*).

### (c) 2D lateral vertex models

A first 2D lateral vertex model was proposed in a seminal work to study the folding and invagination of an epithelium with a locally tubular shape [35]. In this work, cells whose apical length is stretched beyond a certain threshold start to contract. This mechanism can result in the formation of a furrow, which resembles epithelia during the process of neural tube formation and gastrulation (figure 2*c*). *Drosophila* mesoderm invagination, which can be observed in cross-sections of roughly cylindrical embryos, has been a biological system of choice to apply 2D lateral vertex models (figure 2*c*). The folding of the mesoderm in the *Drosophila* embryo has been suggested to result from the buckling of a homogeneous epithelium under compression, based on lateral vertex model simulations resulting in buckled shapes reminiscent of the invaginating embryo [37]. Alternatively, difference in apical and basal tension in the mesoderm can drive tissue invagination, depending on the passive elastic properties of the cells and on the stiffness of the surrounding material [38,39]. The effect of the basement membrane elasticity on folds in flat epithelia induced by apico-basal tension asymmetry has also been discussed [40] (figure 2*c*). 2D lateral vertex model simulations have also been used to study the folding of an epithelium into the optical cup in mouse embryonic stem cell cultures [36] (figure 2*c*).

### (d) 3D vertex models

Similar to 3D apical vertex models, 3D vertex models have been introduced to study epithelial deformation, with a detailed description of forces generated along the apical, lateral and basal surfaces of cells. In a vertex model reproducing growth of an epithelial vesicle into a tube-like structure by a sequence of cell divisions, the choice of friction on the vertex dynamics influences the shape taken by the grown vesicle [43] (figure 2*d*). In the same framework, the influence of apical surface tension on the smoothness of the apical and basal tissue surfaces was investigated [45] (figure 2*d*). 3D vertex model simulations have been used to show that the formation of cysts in the growing wing imaginal disc of *Drosophila* is driven by increased interface contractility between differently fated cell populations [2] (figure 2*d*). A threefold increase in tension between cells of different fates is sufficient to trigger cyst formation in a 3D vertex model, similar to experimental observations. Tissue bending induced by patterning of a spherical epithelium has also been recently discussed [44,69].

### (e) Mechanical inference

The vertex model framework has been used to infer bulk pressure of cells and interface contractility based on experimental observations of the tissue geometry, by solving the mechanical inverse problem [70–72]. Specific assumptions on the mechanics of the tissue must be made to extract mechanical information from geometrical observations. Assuming mechanical equilibrium on vertices hence imposes a set of algebraic constraints, which can be solved to obtain the required mechanical parameters. In a related approach, the forces acting inside an epithelium are inferred from the observed dynamics of the tissue deformation [73]. Active forces generated inside the cells are balanced by passive forces due to viscous dissipation, and mechanical parameters are obtained by solving the balance equations between these forces. Methods previously used to infer forces in two dimensions were recently generalized to 3D [74].

## 4. Towards a continuous approach by large-scale coarse graining

**Figure 3.**
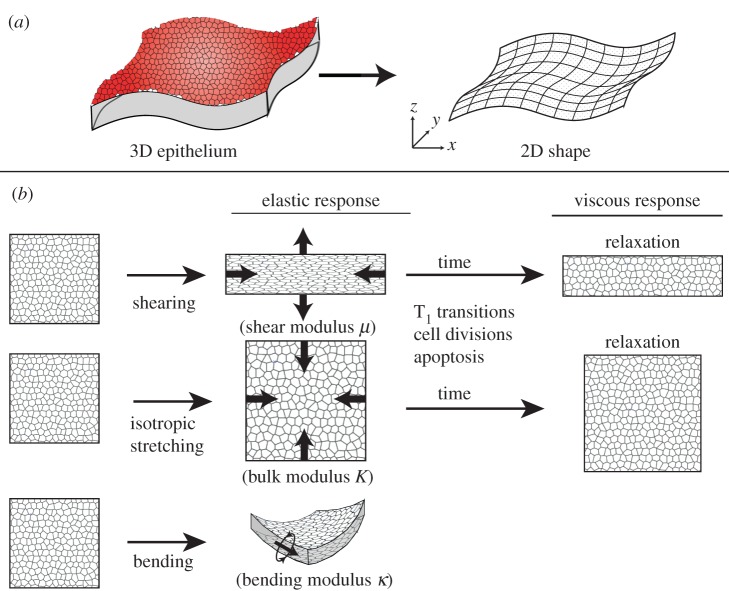
Continuum approaches by coarse graining of vertex models. (*a*) In a continuum theory, the epithelium is represented by a 2D viscoelastic sheet [2,11,33,75]. (*b*) The shear modulus *μ*, the bulk modulus *K* and the bending modulus *κ* characterize the resistance to shear, isotropic stretching and bending of a thin elastic sheet in response to forces acting on the material. On timescales on which cellular neighbour exchange, cell division and cell delamination occur, in-plane isotropic and anisotropic stresses relax and the tissue exhibits a fluid behaviour.

Vertex models are useful in predicting cellular shapes in the tissue. However, in situations involving large-scale tissue flows and deformation, it can be appropriate to describe the tissue with large-scale fields, such as the velocity or cell deformation, that are averaged over lengths larger than the typical cell size (figure 3*a*). Such approaches have the advantage of being generic: different underlying microscopic models can give rise to identical continuum representations [76]. We now briefly discuss attempts at deriving continuum theories from existing vertex models.

On a large-scale, elastic coefficients can be associated with the resistance of a tissue to different modes of deformation (figure 3*b*). The shear modulus *μ* of an epithelium characterizes the response of a tissue to an area conserving shear deformation, and the bulk modulus *K* describes the response to an isotropic area expansion of the tissue. On a longer timescale, cellular rearrangements [11,77], the sensitivity of division to stress [78], as well as pressure dependent cell extrusions [52,54], can lead to the relaxation of planar elastic stresses and to tissue flows (figure 3*b*). In that case, the resistance of the tissue to flows is characterized by viscosities. When an epithelium is allowed to deform in three dimensions, the corresponding continuum theory represents the tissue as a thin curved sheet, and additional coarse-grained parameters describe the response of the tissue to out-of-plane deformations (figure 3*a*). The resistance to bending of the tissue, for instance, is described by a bending modulus *κ*, and the tissue can also have a spontaneous curvature *C*_0_ [2,75].

Vertex model representations can be used to derive coarse-grained mechanical properties of epithelia as a function of the cell shapes and mechanical parameters, assuming that the cellular packing lies on a regular lattice. The bulk and shear modulus of a 2D tissue represented by an apical vertex model can be related to the apical line tensions and area elasticity of the cells [1,33]. In addition to bulk and shear modulus, a bending modulus has been derived in a 3D apical vertex model, after introducing a term that penalizes differences of orientations of adjacent apical cell surfaces [33]. A linear stability analysis of the resulting continuum theory can then be used to study the buckling behaviour of a piece of tissue under compression. Similarly, in a 3D vertex model, the cell shape, bulk and shear moduli, the preferred curvature and bending modulus of an epithelium can be related to active tensions generated along the apical, basal and lateral interfaces of cells [2,75]. The resulting elastic theory can be used to make generic predictions about the 3D vertex model's large-scale behaviour. While these methods can allow the large-scale vertex model elastic behaviour to be connected to local cell parameters, obtaining expressions for coarse-grained elastic coefficients in disordered packings has not been achieved yet.

Although elastic behaviour can be characterized using regular lattices, taking into account topological transitions in the large-scale description of a tissue is an additional challenge. Different approaches have been recently proposed to take into account the effect of topological transitions on large spatial scales [11,79–81]. A key idea in these approaches is to separate the tissue deformation into an average cell deformation and a shear induced by topological changes in the tissue. Although methods can be derived to obtain such a decomposition from experimental data of deforming tissues, it would be interesting to see whether a systematic coarse-graining of vertex models can be achieved, taking into account topological transitions.

## 5. Discussion

Vertex models have been successfully applied to study a variety of phenomena in the field of developmental biology and have helped us to understand epithelial morphogenesis in different model organisms. They have contributed to figuring out the key role played by tensions generated at cellular interfaces in tissue mechanics.

Depending on the geometrical representation of the cells, vertex models can be classified into 2D and 3D apical vertex models, 2D lateral vertex models and 3D vertex models. Apical vertex models are appropriate for understanding cell rearrangements in flat epithelia when forces are essentially generated parallel to the apical surfaces of the cells. 2D lateral vertex models can be used when the main forces act to deform the tissue in the plane of the cross-section, the tissue shape is approximately invariant under in-plane translation, and topological rearrangements do not play a role. 3D vertex models can be applied in a larger class of situations.

Coarse-graining approaches of vertex models are starting to be developed to elucidate the morphogenesis and shapes of epithelia on larger scales. While vertex models describe the shape of the epithelium with cellular details, analytical predictions are difficult to obtain and the model specification can potentially involve a large number of parameters. Continuum approaches depend less on the details of stress generation at the cellular level and can sometimes yield analytical solutions, allowing characterization of the generic behaviour of the tissue. The combination of the detailed representation of the epithelia in vertex models and the analytically tractable continuum representation can therefore help elucidate different facets of tissue mechanics.

Vertex models will most probably play an important role in the future to gain a deeper understanding of how forces generated inside cells affect the shape and mechanics of epithelial sheets. The appearance of new experimental methods that allow for imaging of epithelial morphogenesis with high resolution in time and space will open new frontiers in the application of vertex models to relevant biological questions. These developments could allow for closer quantitative comparisons between simulations and experiments, and estimates of the parameter values of vertex models. A more precise determination of dissipative processes dominating in tissues could also be achieved, in particular by determining the relative role of internal viscous dissipation and external friction opposing tissue motion. Refined imaging techniques could also allow resolution of the details of shape fluctuations on the level of cells, and the corresponding question of the nature and the importance of noise could be addressed in future vertex model studies.

In addition to laser cutting experiments which have become prevalent in recent years [22,25,30,82–84], direct mechanical manipulations [48,85] can be used to probe the mechanical assumptions made in the vertex models and to assess if the chosen parameters are reasonable.

It will also be interesting to further explore the importance of forces generated along the lateral and basal interfaces of cells in epithelia. First steps have been taken towards the quantification and modelling of deforming epithelia in 3D, and the knowledge acquired about the analysis of flat epithelia can further be used to address open questions about the role of the third dimension. 3D vertex models would be especially suited to study processes such as folding, buckling and flattening. As 3D studies become possible, it will also be interesting to explore in more detail the mechanical interplay between epithelia and the extracellular matrix. These pursuits require a multidisciplinary approach and close collaborations between experimentalists and theoretical biophysicists.
